# Contamination of Pet Food with Mycobiota and *Fusarium* Mycotoxins—Focus on Dogs and Cats

**DOI:** 10.3390/toxins12020130

**Published:** 2020-02-19

**Authors:** Natalia Witaszak, Agnieszka Waśkiewicz, Jan Bocianowski, Łukasz Stępień

**Affiliations:** 1 Institute of Plant Genetics, Polish Academy of Sciences, 60-479 Poznań, Poland; lste@igr.poznan.pl; 2 Department of Chemistry, Poznań University of Life Scienses, 60-625 Poznań, Poland; agnieszka.waskiewicz@up.poznan.pl; 3 Department of Mathematical and Statistical Methods, Poznań University of Life Sciences, 60-637 Poznań, Poland; jan.bocianowski@up.poznan.pl

**Keywords:** pet food, *Fusarium*, ergosterol, mycotoxins, trichothecenes, fumonisin B_1_, zearalenone, HPLC

## Abstract

A wide range of pet food types are available on the market; the dominant type is dry food formulated in croquets. One of the most common ingredients of dry food are cereals—vectors of harmful mycotoxins posing the risk to pet health. In this study, 38 cat and dog dry food samples available on the Polish market were investigated. Morphological and molecular methods were applied to identify fungal genera present in pet food. Quantification of ergosterol and *Fusarium* mycotoxins: Fumonisin B_1_, deoxynivalenol, nivalenol, and zearalenone were performed using high performance liquid chromatography. Obtained results indicated five genera of mycotoxigenic fungi: *Alternaria* sp., *Aspergillus* sp., *Cladosporium* sp., *Penicillium* sp., and *Fusarium* sp., including *Fusarium verticillioides* and *Fusarium proliferatum.* Ergosterol and mycotoxins of interest were detected in both cat and dog food samples in the amounts ranging from 0.31 to 4.05 µg/g for ergosterol and 0.3–30.3, 1.2–618.4, 29.6–299.0, and 12.3–53.0 ng/g for zearalenone, deoxynivalenol, nivalenol, and fumonisin B_1_, respectively. The conclusion is the presence of mycotoxins in levels much lower than recommended by EU regulations does not eliminate the risk and caution is advised concerning that long-term daily intake of even small doses of mycotoxins can slowly damage pet’s health.

## 1. Introduction

Companion animals play a significant role in people’s lives since their domestication. In the past they were treated as workers or hunters and now people call them “family members” and “friends”. People often assign human emotions, behavior, and personality traits to animals. This phenomenon is called anthropomorphism [1]. There are numerous research reports available concerning health and emotional benefits coming from the companionship of pets. For example, the presence of a dog or cat in a human’s lifetimes decreases blood pressure and heart rate and reduces stress. Pets can also support treatment during depression and other psychic and emotional disorders [2]. It has been proven that taking care of animals by children teaches them responsibility, builds self-confidence, and ensures appropriate emotional development [3].

Increasing attention and care for pets’ health and well-being is observed because of the relationship between human and companion animals. Therefore, the owners pay heed to diet for their pets more often than they did in the past and this became easier because of a large availability of pet food—a type of animal feed dedicated for companion animals. The pet food industry offers a wide range of food products for dogs and cats, especially wet and dry food. According to the statistical data on statista.com, the mean value of pet food sales in years 2010–2016 was 8.5 million tons per year. It is quite obvious why consumers choose commercial pet food—it is a simple, fast, and cheap way to obtain balanced and differential food for dog or cat [4]. Because dogs are omnivores and cats are carnivores, every food must have a suitable composition and nutrient value. This difference entails consequences in the varying nutrient requirements of these animals. For example, cats use proteins as a main source of energy so they food should contain more proteins (>26%) comparing to dog food (18–22%) according to recommendations of the Association of American Feed Control Officials. Moreover, cat food must include higher doses of taurine, vitamin A, arachidonic acid, and arginine [5]. On the other hand, a dog’s genome contains genes responsible for starch digestion and glucose absorption [6], while cats do not have these genes and are generally not able to digest dietary fiber, which may eventually be fermented by gut bacteria [7,8]. The excess of carbohydrates derived by cereals in dry cat food might affect the animal health in opposition to cooked starch, which is efficiently digested by cats [8].

The main types of pet food are home-cooked and raw food as well as commercially available dry, canned, and semi-moist food. Animal derivatives or by-products are the most important ingredient in dry food followed by vegetables and cereals, which are the main source of starch and, therefore, they play a role of fillers improving croquet consistency. Maize, wheat, rice, and barley are most commonly used to the production of dry pet food. In grain-free pet food cereals are replaced by potatoes and beet pulp. Besides the impact on pets’ gastrointestinal tract (changes in commensal microflora populations), cereals are among the most important sources of toxic contaminations of the pet food [7].

Mycotoxin-producing fungi from the *Fusarium* genus are the primary cereal pathogens, causing every year yield losses world-wide. The most common pathogens of maize are *Fusarium verticillioides* and *F. proliferatum*, while *F. culmorum*, *F. avenaceum*, and *F. graminearum* are common wheat pathogens. Three groups of mycotoxins produced by *Fusarium* fungi are of major importance: fumonisins, trichothecenes and zearalenone, causing sphingolipid metabolism disruption; replication, transcription and translation disturbances and estrogen receptors overexpression, respectively [9]. Only a few research studies are available dealing with the toxicity of mycotoxins to dogs’ and cats’ health. According to data contained in EFSA 2017 report, there are only eight reports about deoxynivalenol (DON) toxicity on pets’ health [10]. Trichothecenes (especially deoxynivalenol) cause appetite and weight loss, diarrhea, vomiting and even gastrointestinal hemorrhage [11,12]. Zearalenone (ZEN) induces oviducts and uterus hyperplasia and edema in bitches and reduces spermatogenesis in dogs [13]. There are no publications about fumonisins affecting companion animals’ health.

During pet food processing dry foods are subjected to extrusion under pressure of 34–37 bars and temperature of 100–200 °C. This process, lowering the moisture to about 8–10% and hermetic storage are common practices used to protect the pellet from contamination and mold development [14,15]. However, this issue does not solve the problem of mycotoxins, which are thermostable and still pose a health risk. Many research reports state food and feed quality problems and their influence on human and animal health but there is limited information about pet food quality and the effect of mycotoxins on their health [16,17,18]. There are enormous numbers of publications and websites about pet nutrition but only few papers about pet food quality, and dog food is more often concerned than cat food. Main health institutions in Europe, USA, and Canada are aware that *Fusarium* mycotoxins pose a health risk. Therefore, there are established regulations of their permissible content in pet food and feed (especially for farm animals) [19,20]. Unfortunately, they still do not apply to the food for dogs and cats. The aim of this study was to investigate dry food for dogs and cats in respect to their microbiological quality and contamination by mycotoxins to estimate its purity and possible risk for companion animals’ vigor.

## 2. Results

**Figure 1:**
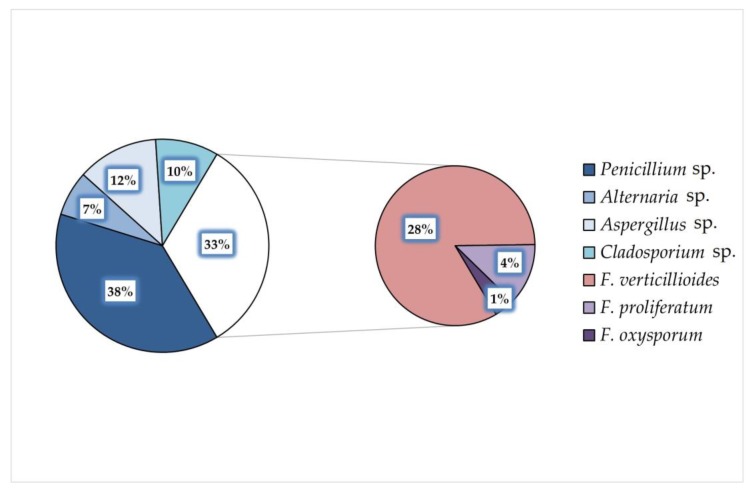
Frequencies of fungal genera and *Fusarium* species isolated and identified in dry pet food samples for cats and dogs.

Pet food producers commonly apply cereals to dry pet food as fillers for croquets consistency improvement. According to the cereal additions’ information, five groups of pet food samples: “maize”, “maize and wheat”, “wheat”, “no cereals”, and “no data” were distinguished. Dog food samples represented all five groups while only three groups were present for cat food because of the lack of samples containing solely wheat and no cereals. Each sample was screened for the presence of filamentous fungi as well as ergosterol content and main *Fusarium* mycotoxins were analyzed (Table 1). Five genera of fungi were identified: *Alternaria* sp., *Aspergillus* sp., *Cladosporium* sp., *Fusarium* sp., and *Penicillium* sp. *Penicillium* sp. was the most common genus contaminating food samples (38%), followed by *Fusarium* sp. (33%) (Figure 1). *F. verticillioides* was commonly observed among *Fusarium* population but *F. proliferatum* and *F. oxysporum* were also identified. *F. verticillioides* and *F. proliferatum* produce fumonisin B_1_, which was detected in eleven samples out of 33 dry pet foods for dogs and cats, regardless of cereal component in the pet food, primarily in samples containing wheat or maize and wheat.

Among all material collected, only two samples did not contain any fungal contaminant, however, the indicator of fungal biomass–ergosterol (ERG) was detected (Table 1). Both samples belonged to the dog food group, and one of them (dF59) still contained fungal mycotoxins. This indicates the need of supporting the mycotoxins investigation with microbiological screening of the samples.

The recommended maximum allowed concentration of ergosterol in grain is 7 μg/g [21]. In our study, ergosterol was detected in all investigated samples and the range of its concentration was 0.31–4.15 μg/g which means that the legal limit has not been exceeded.

Zearalenone, trichothecenes and fumonisin B_1_ were also found but these mycotoxins were not present in all investigated samples. The minimum and maximum values of ZEN, DON, NIV, and FB_1_ were 0.3–30.3, 22.2–618.4, 29.6–299.0, and 17.9–53.0 ng/g, respectively. Deoxynivalenol was the most abundant mycotoxin among all food samples (74%) and it also prevailed in dog food (77%), while zearalenone was dominating in cat food samples. The least frequent mycotoxin was FB_1_, which is very interesting finding, considering the fact that the most frequent isolated species were *F. proliferatum* and *F. verticillioides—*main producers of fumonisin B_1_. In addition, *Fusarium* representatives biosynthesizing trichothecenes and zearalenone, e.g., *F. culmorum*, *F. graminearum*, or *F. equiseti*, were not detected.

Based on cereal composition, three groups were distinguished among cat food samples—food containing: maize and wheat, only maize and samples without composition data (Table 2). The highest mean concentration of ergosterol occurred in cat maize-containing food, and it reached 2.01 µg/g. In samples without the information about cereal composition, significantly different values of the three mycotoxins were observed compared to the samples with maize or wheat and maize. The following concentrations of zearalenone, deoxynivalenol and nivalenol were measured: 17.3, 260.3, and 128.2 ng/g, respectively. In samples containing maize and wheat, nivalenol was not detected. It is worth highlighting that the concentrations of fumonisin B_1_ in cat food with maize were lower than in food containing maize and wheat. Similar trend was observed for dog food but probably relating to a larger set of samples rather than higher concentrations of FB_1_ in food composed with maize and wheat comparing to food with pure maize.

In contrast to cat food samples, in dog food the highest concentrations of ERG and mycotoxins were detected in the samples containing either maize or maize and wheat (Table 3). Examined material presented the highest mean values for trichothecenes (226.4 and 124.8 ng/g for DON and NIV, respectively) in samples containing maize. In turn, dog food with maize and wheat contained maximum concentrations of ZEN (10.9 ng/g) as well as FB_1_ (42.9 ng/g). It is worth noting that dog food sample without cereals contained the lowest level of ergosterol (837.6 ng/g) and zearalenone, nivalenol and fumonisin B_1_ were not detected. Only deoxynivalenol was present in a very low amount—30.9 ng/g.

**Figure 2:**
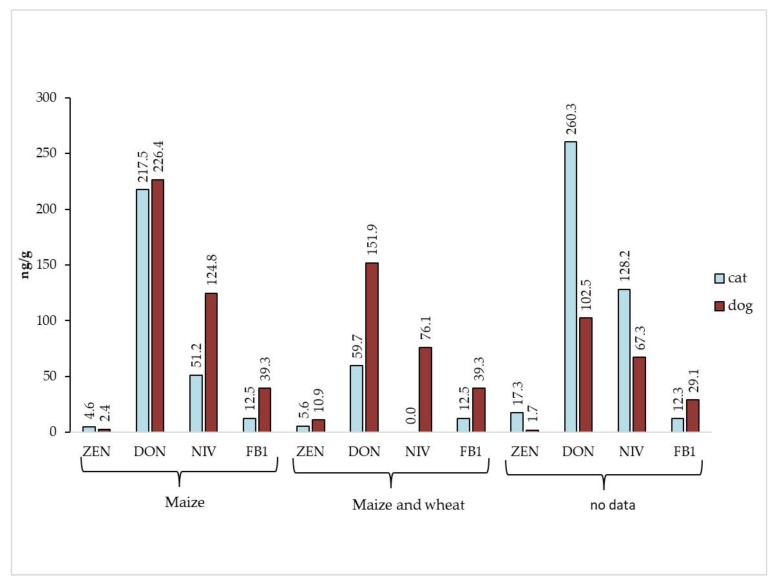
Comparison of *Fusarium* mycotoxins content in cat and dog food samples in relation to their cereal composition.

The comparison of food samples for dogs and cats distinguished two trends in mycotoxins distribution (Figure 2). In the first one, dog food samples with maize/maize and wheat revealed higher concentrations of all mycotoxins (except ZEN) than cat food samples with those cereals. The reverse trend was observed in the samples without cereal addition data—mycotoxin levels in cat food were higher comparing to dog food. The only exception was FB_1_ which level was higher in dog food than in cat food just like in cereal-containing samples.

**Figure 3:**
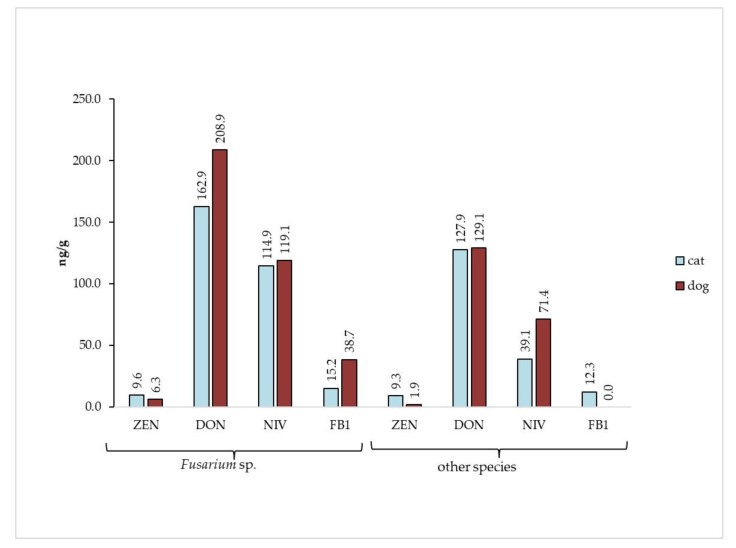
Comparison of *Fusarium* mycotoxins content in cat and dog food samples in relation to their *Fusarium* sp. content.

Only 58% of samples contained detectable *Fusarium* spp. among collected material, while *Fusarium* mycotoxins were detected in 94% of samples. We compared the samples in terms of *Fusarium* species content and their mycotoxins contribution (Figure 3). As expected, the concentrations of mycotoxins were higher in food samples containing *Fusarium* sp. than in food samples with other fungal contaminants. Only one mycotoxin (fumonisin B_1_) was not detected in the sample with the absence of *Fusarium* sp.

So far, no mycotoxin limit values have been established for pet food. In 2006, the European Commission issued a recommendation regarding the permitted levels of mycotoxins in animal feed products [19,20]. The guidance values of DON, ZEN and FB_1_ + FB_2_ in feed with a moisture content of 12% were as follows: 0.9–12, 0.1–3 and 5–60 mg/kg, respectively. Based on these regulations we could conclude that none of the investigated samples exceed the recommended level of mycotoxins.

The analysis of correlation proved high significant correlations between ergosterol and mycotoxins content in dog food samples (with the exception of ZEN) (Table 4). Similarly, high correlations were detected in the case of ZEN/FB_1_ as well as DON/NIV. NIV/FB_1_ and DON/FB_1_ showed significant and high significant correlations, respectively. In turn, in the case of cat food samples only one significant correlation was observed, i.e. between ERG and ZEN. Concentrations of DON and ZEN were strongly correlated.

## 3. Discussion

Pet food industry is a developing area of world economy. According to the data reported by statista.com, there were about 8.5 × 10^4^ of dogs and over 1 × 10^5^ cats in the European Union in 2018 [4]. Different types of pet food are available on the market but dry and canned food are prevailing. In canned food the main ingredient is meat while dry food contains mainly cereal grains. Additives of cereals (mainly rice, wheat and maize) improve pulp consistency and facilitate formulation of croquettes. Unfortunately, most cereals used for pet food production contain pathogens belonging to the *Fusarium* genus, which are responsible for biosynthesis of strongly toxic secondary metabolites (mycotoxins).

Mycotoxins are present in pet food since these compounds have stable chemical structures and are insensitive to most manufacturing processes like cleaning, milling, and boiling [15]. During pet food production manufacturers apply mainly heating and extrusion processes and several studies were conducted concerning the influence of food processing on mycotoxins’ concentrations. It has been shown that high temperature baking as well as extrusion are the most relevant in the case of mycotoxins content reduction, however, the effectiveness of these processes depends on conditions used, like temperature, duration of the process, or humidity. The decrease of mycotoxins concentrations during different types and conditions of food processing ranged between 20–65%, 23–92%, 24–70% for FB_1_, ZEN, and DON, respectively [22]. Taking into account the degradation of mycotoxins during extrusion, it is possible that the concentrations of mycotoxins included in the investigated samples are lower than in the grain used for pet food production. Simultaneously, it can be stated that efficiency of these processes is not satisfactory in the light of fast progressing climate change. Forecast for Europe predicts climate warming and rising humidity, thus, better conditions for *Fusarium* sp. development and, eventually, extensive mycotoxin contamination and reduction of yield and quality are expected [22,23,24,25].

Mycotoxigenic fungal genera were identified in tested pet food samples. Both storage genera, like *Penicillium* sp. (38%) and *Aspergillus* sp. (12%), as well as plant-derived *Fusarium* sp. (33%) were identified. This result confirms the findings reported by previously published papers [26,27,28,29]. Interesting observation is the presence of *Fusarium* sp. responsible for biosynthesis of fumonisin B_1_ (*F. proliferatum, F. verticillioides*) only, while zearalenone and trichothecenes produced mainly by *F. graminearum* and *F. culmorum* were also detected. Possible explanation of this phenomenon is the fact that *F. graminearum* and *F. culmorum* spores and hyphae are more sensitive to high temperatures and were inactivated during the manufacturing process. It is worth emphasizing that *Aspergillus* sp. was identified in collected material. This might result in the presence of aflatoxins, so the analysis of these highly harmful mycotoxins would bring the broader context into mycotoxicological research of pet food.

Most studies on pet food contamination with mycotoxins were focused on single group or a narrow range of mycotoxins [11,30,31,32,33,34,35], but currently the studies are focused on comprehensive approach, as demonstrated by Bohm et al. [11] and Gazzotti et al. [33], who analyzed *Fusarium* mycotoxins: Fumonisin B_1_, deoxynivalenol, and zearalenone [34,35]. In our research FB_1_, DON, and NIV, as well as ZEN, were quantified in both dogs’ and cats’ food. The presence of mycotoxins of interest in feed material was expected and the most frequent compounds were DON and ZEN. The surprising result is the higher concentrations of FB1 observed in both cat and dog food containing maize and wheat than in those with maize only, which is regarded a main vector for this mycotoxin. In general, wheat is considered free of fumonisins because it is not a host-plant for fumonisins-producing *Fusarium* species (e.g., *F. proliferatum, F. verticillioides*), but some recent reports revealed this mycotoxin in wheat and wheat by-products [36,37]. According to a study conducted by Chehri et al. [38] *F. verticillioides* was not only detected in wheat grain samples but it was also the second most frequent species after *F. graminearum*. Nevertheless, concentration values of investigated mycotoxins in pet food were below the acceptable levels recommended by the EU regulations. Our results were in accordance with the data obtained during a long-term international research on mycotoxins in feed [25]. According to these authors up to 88% of samples were contaminated by at least one of the mycotoxins but in Europe feed contamination did not exceed the permitted level. The same study indicates the *Fusarium* mycotoxins—DON and ZEN—as one of the most significant feed contaminants.

Tegzes et al. tested 60 samples of grain and grain-free dog food for aflatoxins, ochratoxin A as well as *Fusarium* mycotoxins contamination [34]. On the contrary to the previous studies, aflatoxins and ochratoxin A were not detected, which has been explained by the effectiveness of quality management regulations. Furthermore, it might be concluded that cereal additives are the main source of mycotoxins contamination because grain-free samples were not contaminated by these compounds. Our study reveals that dog and cat foods without cereals are generally free of mycotoxins; however, very low levels of DON (21.0–42.2 ng/g) were detected.

Recent studies increasingly indicated that high resolution mass spectrometry is a powerful tool in a large scale of mycotoxins screening as well as food and feed comprehensive analyses [35,39]. Castaldo et al. examined 89 pet food samples, which then were subjected to a targeted analysis of 28 most common mycotoxins, i.e., *Fusarium* mycotoxins, aflatoxins and ochratoxin A [35]. Then, a retrospective analysis was performed resulting in the detection of 245 different toxic bacterial and fungal metabolites, among which still *Fusarium* mycotoxins were the most common. Another problem which has been highlighted by this paper, is the presence of about 16 toxins per sample and their possible implication on animal health [25]. The interactions between mycotoxins can be synergistic, additive, less than additive and antagonistic. The possible implications of mycotoxins’ synergism were summarized in extensive review article by Greiner and Oswald [40]. Results of a large-scale research by Gruber-Dorninger et al. proved that the presence of few different mycotoxins is a general rule so the risk of affection of human and animals’ health’s through synergistic action of different mycotoxins seems to be high [25]. Our studies revealed the co-occurrence of *Fusarium* mycotoxins as well, and it seems to be natural because of the ability of species to produce more than one mycotoxin (for example *F. proliferatum* is able to produce fumonisins, moniliformin, and beauvericin) [41]. Moreover, food and its by-product might be contaminated by different fungi during product lifetime (on the field, during storage) and fungal secondary metabolites can be accumulated in it. At this moment, a global list of feed and food contaminants as well as cumulative effect of these toxins is unknown, hence this is the future perspective in feed and food research. Both studies show the results similar to those obtained in the present study, thereby confirming the hypothesis that *Fusarium* mycotoxins are present in pet food and threaten pets’ health. Moreover, it has been proven that the topic of food contamination by these mycotoxins is still alive and no crucial solution has been applied so far. Despite the fact that the concentrations of chosen mycotoxins did not exceed the permitted levels established by EU, it does not mean that pets’ health is not in danger. Dry food is mostly a basis of pet diet and even extremely low doses of mycotoxins might result in chronic diseases when consumed daily. Mycotoxins could be ingested by the animal with contaminated food and accumulate in their tissues or excrete with body fluid, hence, meat, milk, and eggs might be also potentially an origin of mycotoxins in diet [42,43,44,45]. It might be concluded that multi-exposure to mycotoxins is an inevitable phenomenon.

Our previous study was dedicated to veterinary diets for dogs and cats. It has shown that these products contain molds as well as *Fusarium* mycotoxins [46]. Detected levels of DON, ZEN, and FBs were much below FDA recommendations but in this particular case it does not guarantee safety. Veterinary diet is a special purpose food which is applied during the treatment of sick animal, possibly more susceptible to harmful mycotoxins. This suggests that the issue covers also other pet food products and, hence, research should be extended to include other types of pet food.

## 4. Conclusions 

After years of research on *Fusarium* mycotoxins in food and feed—especially pet food—the knowledge is still insufficient. It is necessary to find the effective solutions on how to reduce mycotoxins in feed product to protect human and animal lives. Limitation of grain used for pet food production might significantly reduce *Fusarium* mycotoxins presence. On the other hand, introduction of new standards in quality assurance based on high resolution mass spectrometry techniques gives a possibility of comprehensive mycotoxin detection as well as their by-products, and effective monitoring and management of mycotoxin contamination.

## 5. Materials and Methods 

**Figure 4:**
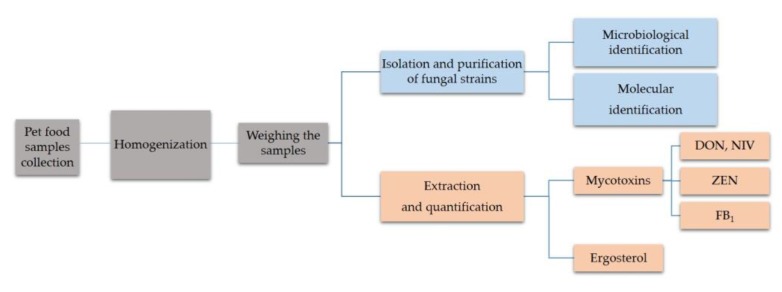
Scheme of sampling of cat and dog food and design of the experiment.

Studied material was collected from hermetically sealed packages of dry food for dogs and cats of 16 producers. They were obtained from pet shops and veterinary clinics in Poland. In total, the collected research material was represented by 38 samples including 12 foods for cats and 26 for dogs. The weights of the samples ranged from 60 to 150 g and the moisture was from 3.8% to 7.1%. Cereals composition was used as division criterion and the following groups were made: (i) Samples containing only maize, (ii) wheat, (iii) maize and wheat, and (iv) none of these cereals. According to this criterion we obtained 3 groups of cat food and 5 groups of dog food (Table 1). Samples of food formulated in differently-sized croquettes were homogenized before the analysis using liquid nitrogen and ceramic mortar, and then milled. Samples were stored at −20 °C until analysis (Figure 4).

### 5.1. Identification of Fungal Isolates

Identification of fungi was performed using microscopic and molecular methods. Genera of fungi were determined on morphological basis and isolates belonging to *Fusarium* genus were then subjected to molecular analyses to obtain species identification.

Two microbiological media: Synthetic nutrient agar (SNA) and potato dextrose agar (PDA) were used for microbiological analysis. First, about 1 g of milled food samples was incubated for one week at room temperature on the PDA medium. Pure fungal isolates were obtained through multiple passaging onto fresh PDA media and then transferred to the SNA medium. The plates were incubated at room temperature for 4 weeks until the appearance of spores. Macrospores were viewed microscopically at 200× magnification and their morphological features were used for genus identification.

Molecular methods were applied only for *Fusarium* isolates. At first, the mycelia were frozen in liquid nitrogen and homogenized using plastic mortar. Then, genomic DNA was extracted according to the CTAB method [47].

The next step was setting up the polymerase chain reaction (PCR) which was performed using Tef1R and Ef728M primers and Taq DNA polymerase (Thermo, Waltham, MA, USA). The quality of PCR products was evaluated based on electrophoretic separation. Subsequently, PCR products were purified with exonuclease I (Epicentre, Madison, WI, USA) and alkaline phosphatase SAP (Promega, Madison, WI, USA). Fluorescent labeling was performed using Ef728M primer and BigDyeTerminator v 3.1 Cycle Sequencing Kit (Applied Biosystems, Foster City, CA, USA).

Sequence reading was done by Laboratory of DNA Sequencing and Oligonucleotide Synthesis at Institute of Biochemistry and Biophysics, Polish Academy of Sciences, Warsaw, Poland. Molecular identification of fungal species was done by comparing the sequences to the reference sequences deposited in the NCBI GenBank database using the BLASTn algorithm. The method was described in detail by Witaszak et al. [46].

### 5.2. Standards and Chemical Reagents

Ergosterol (ERG) and mycotoxins (fumonisin B_1_—FB_1_, zearalenone—ZEN, deoxynivalenol—DON, and nivalenol—NIV) standards were purchased with a standard grade certificate (purity above 98%) from Sigma-Aldrich (Steinheim, Germany). Stock solutions of standards were prepared in acetonitrile (except ERG in methanol) at 1.0 mg/mL concentrations and stored at −20 °C. Sodium dihydrophosphate, potassium hydroxide, sodium hydroxide, potassium chloride, acetic acid, hydrochloric acid, and o-phosphoric acid were purchased from POCh (Gliwice, Poland). Organic solvents (HPLC grade), disodium tetraborate, n-pentane, 2-mercaptoethanol, sodium acetate, and all the other chemicals were also purchased from Sigma-Aldrich (Steinheim, Germany). Water for the HPLC mobile phase was purified using a Milli-Q system (Millipore, Bedford, MA, USA).

### 5.3. Extraction and Purification Procedure

#### 5.3.1. Ergosterol 

Samples (each in triplicate) containing 100 mg of ground material were suspended in 2 mL methanol in a culture tube and treated with 0.5 mL of 2 M aqueous sodium hydroxide. Samples were irradiated twice in a microwave oven (370 W) for 20 s according to Waśkiewicz et al. [48]. After 15 min contents of cultures tubes were neutralized with 1 M aqueous hydrochloric acid, then 2 mL of methanol were added and samples were extracted with pentane (3 × 4 mL). The combined pentane extracts were evaporated to dryness in a stream of nitrogen, before analysis dissolved in 1 mL of methanol and 10 µL of thus prepared mixture were analyzed by HPLC.

#### 5.3.2. Mycotoxins

Ground samples (10 g) were soaked with 30 mL of acetonitrile:water (80:20, *v*/*v*) and mycotoxins (DON, NIV, ZEN) were extracted overnight. After filtration (Whatman No. 5 paper, Whatman International Ltd, Maidstone, UK), 10 mL of the extract was collected and diluted with 40 mL of water. Half of the extract was used for DON and NIV analysis, while the second part was used for ZEN determination. In both cases, the extract was applied on top of an immunoaffinity DON-NIV column and the Zearala Test column (IAC, Vicam, Milford, MA, USA) for DON/NIV and ZEN analysis, respectively in accordance with the manufacturer’s procedure.

Fumonisin B_1_ was extracted from ground samples of veterinary diet (10 g) by homogenization for 3 min in 30 mL of methanol-water (3:1, *v*/*v*) and filtration through Whatman no. 4 filter paper (Whatman International Ltd, Maidstone, UK) according to Stępień et al. [49]. The filtered extract (pH = 5.8–6.3) was applied at the top of the conditioned cartridge (a SAX cartridge, Bond Elut, Agilent Santa Clara, CA, USA) with flow rate of 2 mL/min. Fumonisin B_1_ was eluted from the column with 10 mL of 1% acetic acid in methanol. The elute was evaporated to dryness at 40 °C under a stream of nitrogen. Dry residue was stored at −20 °C until HPLC analyses.

### 5.4. HPLC Analysis 

The chromatographic system consisted of Waters 2695 high-performance liquid chromatograph (Waters, Milford, USA) with detectors: (i) Waters 2996 Photodiode Array Detector with Nova Pak C−18 column (150 × 3.9 mm) and methanol:acetonitrile (90:10, *v*/*v*) as a mobile phase for ERG (λ_max_ = 282 nm) analysis and with Nova Pak C−18 column (300 × 3.9 mm) and methanol:water (25:75, *v*/*v*) as a mobile phase for DON and NIV analysis (λ_max_ = 224 nm); (ii) Waters 2475 Multi λ Fluorescence Detector (λ_ex_ = 274 nm, λ_em_ = 440 nm) and Waters 2996 Photodiode Array Detector with Nova Pak C−18 column (150 × 3.9 mm) and acetonitrile:water:methanol (46:46:8, *v*/*v*/*v*) as a mobile phase for ZEN analysis; (iii) Waters 2475 Multi λ Fluorescence Detector (λ_ex_ = 335 nm, λ_em_ = 440 nm) with an XBridge column (3.0 × 100 mm) and methanol:sodium dihydrogen phosphate (0.1 M in water) solution (77:23, *v*/*v*) adjusted to pH 3.35 with o-phosphoric acid as a mobile phase for FB_1_ analysis after pre-column derivatization with *o*-phthaldialdehyde (OPA) reagent.

The linearity of the standard curves was 0.9897, 0.9991, 0.9990, 0.9989, and 0.9982 for FB_1_, ZEN, DON, NIV, and ERG, respectively. The limits of quantification (LOQ) were (in ng/g): 1.0 for ZEN, 2.0 for FB_1_ and 10.0 for DON, NIV, and ERG. The recovery experiment was performed on mycotoxin-free veterinary diet samples, spiked with three different levels of each mycotoxin separately at a concentration of 5.0, 8.0, and 10.0 ng/g for FB_1_ and ZEN and 20.0, 40.0, and 80 ng/g for DON and NIV. On the basis of these experiments, recovery rates and standard deviations (RSD) were calculated. For the majority of the tested mycotoxins, the recovery rates ranged between 88% and 109% (depending on the matrix), whereas the RSD values did not exceed 13%.

### 5.5. Statistical Analysis

Firstly, the normality of distributions for studied traits was tested using the Shapiro–Wilk normality test [50]. One-way analysis of variance (ANOVA) was carried out to determine the effects of cereal composition on the variability of ergosterol (ERG), and mycotoxins: zearalenone (ZEN), deoxynivalenol (DON), nivalenol (NIV), and fumonisin B_1_ (FB_1_), independently for cat and dog food samples. Minimal, maximal and mean values of individual traits were calculated. The Fisher’s least significant differences (LSDs) were calculated for individual characteristics and on this basis homogeneous groups were determined. The relationships between the ERG, FB_1_, ZEN, DON, and NIV were determined using the simple correlation analysis. Relationships of five observed traits were presented in Table 4. Data analysis was performed with the GenStat 18 package software for cat and dog food samples independently.

## Figures and Tables

**Table 1:** Fungal species and genera isolated and identified in dog and cat food samples, along with ergosterol, zearalenone, deoxynivalenol, nivalenol and fumonisin B_1_ concentrations measured in the respective samples. The samples were grouped based on their cereal component.

Pet	Sample No.	*Fusarium* sp.	Other Fungi	ERG [μg/g]	Mycotoxins			
					ZEN [ng/g]	DON [ng/g]	NIV [ng/g]	FB_1_ [ng/g]
	**maize and wheat**							
cat	cF1	Fv, Fp	P	0.73 ± 0.04	3.9 ± 0.3	55.3 ± 3.6	n.d.	17.9 ± 2.9
	cF2	Fv	P	1.04 ± 0.07	4.1 ± 0.4	104.5 ± 8.6	n.d.	n.d.
	cF4	Fv	P	4.14 ± 0.19	n.d.	22.2 ± 4.6	n.d.	n.d.
	cF15	Fv	P	1.75 ± 0.05	8.7 ± 1.5	53.0 ± 9.4	n.d.	n.d.
dog	dF36	−	P, C	0.88 ± 0.03	2.0 ± 0.5	n.d.	29.6 ± 4.0	n.d.
	dF39	Fv	P, Alt	1.36 ± 0.07	n.d.	n.d.	n.d.	28.9 ± 2.4
	dF40	Fo	P	0.43 ± 0.04	n.d.	n.d.	n.d.	n.d.
	dF42	−	P	0.85 ± 0.05	0.3 ± 0.1	97.3 ± 7.6	n.d.	n.d.
	dF45	Fv	P, C	1.32 ± 0.17	30.3 ± 2.6	303.4 ± 18.9	86.0 ± 13.6	53.0 ± 4.2
	dF46	Fv	P, A	1.19 ± 0.13	n.d.	279.6 ± 18.7	76.9 ± 3.7	46.9 ± 2.3
	dF47	−	P, A	0.50 ± 0.03	n.d.	76.3 ± 3.7	100.1 ± 14.1	n.d.
	dF58	−	P	0.41 ± 0.02	n.d.	129.9 ± 13.6	102.9 ± 9.4	n.d.
	dF59	−	−	0.83 ± 0.02	n.d.	24.6 ± 5.0	61.3 ± 3.2	n.d.
	**maize**							
cat	cF12	Fv, Fp	P, C	0.85 ± 0.04	4.5 ± 0.8	n.d.	42.0 ± 3.1	n.d.
	cF13	−	P	2.04 ± 0.14	2.8 ± 0.2	217.5 ± 18.4	46.7 ± 4.1	n.d.
	cF14	Fv	−	4.05 ± 0.07	2.0 ± 0.2	n.d.	64.8 ± 4.2	12.5 ± 2.9
	cF29	Fv	P, C	1.09 ± 0.14	9.1 ± 2.1	n.d.	n.d.	n.d.
dog	dF33	Fv	P	2.95 ± 0.18	n.d.	554.3 ± 40.4	299.0 ± 25.9	31.9 ± 2.6
	dF51	Fv	P, A	1.18 ± 0.12	3.0 ± 0.3	99.4 ± 8.3	66.1 ± 4.8	33.5 ± 3.6
	dF52	Fv	P, A	0.83 ± 0.04	0.5 ± 0.0	114.7 ± 12.3	n.d.	n.d.
	dF53	Fv	P, A, Alt	1.18 ± 0.07	5.3 ± 0.3	n.d.	n.d.	52.4 ± 2.7
	dF54	−	C, P	0.31 ± 0.02	n.d.	203.1 ± 10.1	n.d.	n.d.
	dF56	Fv	P	0.74 ± 0.04	1.7 ± 0.48	300.1 ± 20.8	n.d.	n.d.
	dF57	−	A	1.58 ± 0.05	3.7 ± 0.3	195.1 ± 9.1	96.5 ± 8.2	n.d.
	dF60	−	P, C	0.78 ± 0.03	1.2 ± 0.3	307.8 ± 15.1	37.7 ± 3.6	n.d.
	dF80	−	A, Alt	1.05 ± 0.08	1.9 ± 0.2	n.d.	n.d.	n.d.
	dF81	−	P, Alt	1.02 ± 0.07	2.1 ± 0.2	36.8 ± 3.7	n.d.	n.d.
	**wheat**							
dog	dF43	Fv	P	1.39 ± 0.12	n.d.	195.4 ± 13.5	n.d.	33.7 ± 3.8
	dF50	−	−	0.38 ± 0.02	n.d.	n.d.	n.d.	n.d.
	dF55	−	P	0.50 ± 0.03	n.d.	195.8 ± 8.8	n.d.	n.d.
	**no cereals**							
dog	dF49	Fp	P, A	0.66 ± 0.04	n.d.	37.3 ± 5.4	n.d.	n.d.
	dF79	−	P, Alt	1.01 ± 0.04	n.d.	24.5 ± 3.4	n.d.	n.d.
	**no data**							
cat	cF24	Fv	−	1.28 ± 0.16	27.2 ± 6.1	618.4 ± 35.1	87.2 ± 8.1	n.d.
	cF25	Fv	A	1.28 ± 0.13	17.2 ± 5.3	124.3 ± 8.2	265.8 ± 21.4	n.d.
	cF26	−	P, C	1.83 ± 0.19	1.6 ± 0.9	38.2 ± 6.4	31.6 ± 3.1	n.d.
	cF30	−	P	0.95 ± 0.06	23.4 ± 5.6	n.d.	n.d.	12.3 ± 2.1
dog	dF48	Fv	−	0.58 ± 0.02	1.2 ± 0.2	112.0 ± 17.3	67.3 ± 4.4	n.d.
	dF78	Fv	A	0.71 ± 0.02	2.2 ± 0.2	93.1 ± 7.7	n.d.	29.1 ± 3.4

cF–cat food, dF–dog food, Fv–F. verticillioides, Fp–F. proliferatum, Fo–F. oxysporum, P–Penicillium, C–Cladosporium, A–Aspergillus, Alt–Alternaria, n.d.–not detected.

**Table 2:** Content of ergosterol and *Fusarium* mycotoxins in cat food samples in relation to their cereal composition.

Cereal Composition	ERG [µg/g]		Mycotoxins [ng/g]							
			ZEN		DON		NIV		FB_1_	
	Range	Mean	Range	Mean	Range	Mean	Range	Mean	Range	Mean
maize	0.81–4.12	2.01 ^a^	1.9–11.3	4.6 ^b^	196.4–230.2 *	217.5 ^b^	43.0–69.0	51.2 ^b^	10.0–15.6 *	12.5 ^a^
maize and wheat	0.70–4.35	1.93 ^a^	3.6–10.3	5.6 ^b^	18.7–112.3	59.7 ^b^	n.d.	n.d. ^b^	15.0–20.8 *	17.9 ^a^
no data	0.90–2.03	1.34 ^a^	0.9–32.2	17.3 ^a^	32.6–652.9	260.3 ^a^	29.2–281.0	128.2 ^a^	10.1–14.4 *	12.3 ^a^
LSD_0.05_		0.94		5.71		134.22		53.07		5.51
ANOVA *F*		0.301		<0.001		0.047		0.003		0.841

n.d–not detected; a, b–in columns, means followed by the same letters are not significantly different; * calculations based on one sample.

**Table 3:** Content of ergosterol and *Fusarium* mycotoxins in dog food samples in relation to their cereal composition.

Cereal Composition	ERG [µg/g]		Mycotoxins [ng/g]							
			ZEN		DON		NIV		FB_1_	
	Range	Mean	Range	Mean	Range	Mean	Range	Mean	Range	Mean
maize	0.29–3.13	1.16 ^a^	0.5–5.6	2.4 ^a^	32.8–593.1	226.4 ^a^	33.6–325.5	124.8 ^a^	29.5–55.5	39.3 ^a^
maize and wheat	0.38–1.45	0.86 ^ab^	0.2–32.6	10.9 ^a^	19.3–324.6	151.9 ^ab^	26.5–114.8	76.1 ^a^	26.5–57.0	42.9 ^a^
wheat	0.36–1.50	0.76 ^ab^	n.d.	n.d.	184.7–210.5	195.6 ^ab^	n.d.	n.d.	29.6–37.1 *	33.7 ^a^
no cereals	0.62–1.06	0.84 ^ab^	n.d.	n.d.	21.0–42.2	30.9 ^b^	n.d.	n.d.	n.d.	n.d.
no data	0.56–0.72	0.65 ^b^	1.0–2.4	1.7 ^a^	86.2–127.1	102.5 ^ab^	62.5–71.1 *	67.3 ^a^	29.6–32.8	29.1 ^a^
LSD_0.05_		0.458		5.22		115.3		56.58		16.88
ANOVA *F*		0.048		0.441		0.043		0.123		0.572

n.d.–not detected; a, b–in columns, means followed by the same letters are not significantly different; * calculations based on one sample.

**Table 4:** Correlation coefficient factor between ergosterol and mycotoxins. Data highlighted in blue represent samples of cat food and in orange—dog food.

Trait	ERG	ZEN	DON	NIV	FB_1_
ERG	1	−0.4239 *	−0.1611	−0.0423	0.0056
ZEN	0.1736	1	0.5604 ***	0.3533 *	0.0148
DON	0.5451 ***	0.2197	1	0.2924	−0.26
NIV	0.6633 ***	0.1228	0.6627 ***	1	−0.197
FB_1_	0.5195 ***	0.4944 ***	0.2968 **	0.2458 *	1

* *p* < 0.05; ** *p* < 0.01; *** *p* < 0.001.
